# Internal quality assurance of HIL indices on Roche Cobas c702

**DOI:** 10.1371/journal.pone.0200088

**Published:** 2018-07-06

**Authors:** Giuseppe Lippi, Janne Cadamuro, Elisa Danese, Matteo Gelati, Martina Montagnana, Alexander von Meyer, Gian Luca Salvagno, Ana-Maria Simundic

**Affiliations:** 1 Section of Clinical Biochemistry, University of Verona, Verona, Italy; 2 Department of Laboratory Medicine, Paracelsus Medical University, Salzburg, Austria; 3 Institute of Laboratory Medicine, Kliniken Nordoberpfalz AG, Weiden, Germany; 4 Institute of Laboratory Medicine, Klinikum St. Marien, Amberg, Germany; 5 Department of Medical Laboratory Diagnostics, University Hospital Sveti Duh, Zagreb, Croatia; Holbæk Hospital, DENMARK

## Abstract

Automatic assessment of hemoglobin (H), lipaemia (L) and icterus (I) in serum or plasma (HIL indices) is the mainstay for evaluating sample quality. We planned this study to verify whether in-house prepared internal quality control (IQC) materials may be suitable for quality assurance of HIL indices. Pools containing different values of each of the three HIL indices were prepared from routine plasma samples, divided in aliquots and frozen at -20°C. Stability of frozen materials was assessed by thawing one aliquot of each pool after different days of freezing (1, 4, 8, 15, 22 and 29), and by measuring HIL indices on baseline fresh samples and frozen-thawed aliquots with Roche Cobas c702. Five fresh liquid IQCs materials were also measured at the same time points. Intra-assay and inter-assay imprecision of HIL indices calculated with commercial IQC materials ranged between 1.1–2.0% and 1.6–3.3%, respectively. When target values of HIL indices were calculated using frozen-thawed aliquots, the inter-assay imprecision of in-house prepared materials was optimal, even better than that of commercial liquid IQCs (H-index, 0.8% versus 1.6%; L-index, 2.2% versus 2.5%; I-index, 0.8% versus 3.3%). In conclusion, in-house prepared IQC materials are cost-effective alternatives to commercial liquid IQCs for HIL quality assurance.

## Introduction

Sample quality is an essential requirement for generating reliable and clinically exploitable results of laboratory testing. Several lines of evidence now attest that the presence of high concentrations of some interfering substances such as cell-free haemoglobin, lipids and bilirubin may impair the quality of testing, causing both biological and analytical bias [1,2]. Albeit identifying the presence of these potentially interfering substances in serum or plasma has been for long carried out by visual inspection, the development and straightforward implementation in most preanalytical, clinical chemistry, immunochemistry and coagulation analyzers of automatic assessment of cell-free hemoglobin (H), lipaemia (L) and icterus (I) in serum or plasma samples (i.e., the so-called HIL indices) has now become the gold standard for evaluating sample quality [3,4], but also for obtaining valuable information about the local blood collection performance [5]. Nevertheless, quality assurance of HIL indices remains an essentially unexplored issue [6].

As any other laboratory test, HIL indices should be subjected to specific requirements by regulatory and/or accrediting bodies [7]. Clinical laboratories should hence define a process of quality assurance for these measures, to be performed along with other clinical chemistry, immunochemistry and hemostasis tests. The HIL indices are typically generated by spectrophotometric assessment using the modern laboratory instrumentation, so that they may be vulnerable to both failures and drifts as any other spectrophotometric test. Albeit no HIL calibrators have become available so far for being used in routine laboratory practice, the Clinical and Laboratory Standards Institute (CLSI) document C56-A [8] has provided general indications that quality assurance of HIL indices should be regularly monitored before using data of these measures for purposes of accepting or rejecting serum or plasma samples before testing. This may be accomplished by using either commercial materials, which are now becoming available [9], or else by in-house preparation of quality control materials derived from routine serum or plasma samples, which can then be frozen and regularly thawed for internal quality control (IQC) assessment. Interestingly, this latter approach not only has been endorsed by Miller et al, who showed that commercial IQCs are not always suitable for verifying consistency of laboratory data, especially when changing reagent lots [10], but may also carry notable advantages over commercial IQC materials, which are mostly attributable to the use of a more uniform sample matrix, a greater commutability and higher recovery, lower costs and potentially enhanced accuracy (i.e., lower chance of errors during manual reconstitution of commercial lyophilized materials) [11,12]. Importantly, the use of commercially available quality controls for monitoring HIL performance has also been recently questioned by Petrova et al, who showed that commercially available cell-free hemoglobin quality control materials displayed poor recovery with H-index assessment, a problem which has been attributed to their potentially unsuitable sample matrix [13].

Although the local preparation of IQC materials seems hence an appealing approach for quality assurance of HIL indices, as recently endorsed by the European Federation of Clinical Chemistry and Laboratory Medicine (EFLM) Working Group for Preanalytical Phase (WG-PRE) [14], little is known about feasibility, validity and effectiveness of this strategy in routine laboratory practice. Therefore, this study was planned to verify whether in-house IQC materials prepared from routine samples may have sufficient stability upon storage at -20°C to be used for regular quality assurance of HIL indices. A secondary endpoint of this study was to investigate the performance of HIL indices assessment using a conventional clinical chemistry analyzer during a nearly 1-month period.

## Materials and methods

Nine different plasma pools, containing increasing values of each of the three HIL indices (low, “L”; medium “M”; high “H” for each HIL index), were prepared from lithium-heparin plasma samples collected in primary evacuated blood tubes (Vacutest Kima, Kima, Arzergrande, Padova, Italy) and referred to the laboratory of the University Hospital of Verona (Italy) for routine clinical chemistry testing (Table 1).

**Table 1:** Values of HIL (Hemolysis, H; Icterus, I; Lipaemia, L) indices measured on fresh lithium-heparin plasma pools on Roche Cobas c702. Values in **bold** are those used for purposes of HIL quality assurance.

Parameter	Roche Cobas c702 arbitrary values					Analyte values
	H-index	L-index	I-index	Hemoglobin (g/L)	Triglycerides (mmol/L)	Bilirubin (μmol/L)
H-index Pool “Low” (L)	**16**	9	16	0.16	-	-
H-index Pool “Medium” (M)	**51**	11	16	0.51	-	-
H-index Pool “High” (H)	**166**	21	13	1.66	-	-
L-index Pool “Low” (L)	2	**10**	19	-	0.8	-
L-index Pool “Medium” (M)	4	**25**	15	-	1.4	-
L-index Pool “High” (H)	7	**386**	11	-	11.8	-
I-index Pool “Low” (L)	3	11	**24**	-	-	13
I-index Pool “Medium” (M)	2	14	**35**	-	-	22
I-index Pool “High” (H)	0	19	**189**	-	-	148

Each plasma pool was divided in 7 identical aliquots of 0.7 mL into 1-mL plastic safe-locks cups. HIL indices were immediately measured on fresh plasma pools, whilst the 6 remaining aliquots of each pool were frozen at -20°C, at dark. Stability of frozen material was then assessed by thawing one aliquot of each pool after different days of storage (day 1, 4, 8, 15, 22 and 29), and by measuring the HIL indices on a Roche Cobas c702 (Roche Diagnostics, Basel, Switzerland), according to manufacturer’s recommendations. Two fresh clinical chemistry IQC commercial materials (Bio-Rad Multi 1 and 3; Bio-Rad Laboratories, Milano, Italy), and the three fresh Liquichek Serum Indices quality controls (“Hemolysis”, “Icterus” and “Lipemia”; Bio-Rad Laboratories, Milano, Italy) were also tested along with the locally prepared IQCs throughout the study period (i.e., day 0, 1, 4, 8, 15, 22 and 29). The specific characteristics of HIL indices assessment using Roche Cobas clinical chemistry platforms have been previously described elsewhere [15,16]. Briefly, the H-index, L-index and I-index are assessed with bychromatic measurements at 570/600 nm, 660/700 nm and 480/505 nm, respectively, and are finally reported in arbitrary units, which can then be converted into concentration of hemoglobin for the H-index (100 = ~100 mg/dL; ~1 g/L), triglycerides for the L-index (100 = ~3.50 mmol/L; ~309 mg/dL), and bilirubin for the I-index (100 = ~74.6 μmol/L; ~4.36 mg/dL). The performance of H-index for estimation of hemoglobin concentration was found to be optimally correlated with the reference cyanmethemoglobin assay [17]. Total cholesterol (enzymatic, colorimetric method) and triglycerides (enzymatic, colorimetric method) were also tested on L-index aliquots, whist total and conjugated bilirubin (quantitative diazo colorimetric assays) were measured on I-index aliquots, using the same Roche Cobas c702 and proprietary reagents (Roche Diagnostics).

All commercial and in-house prepared IQC materials were measured in duplicate and the final value was expressed as the mean of the two replicates. The same analyzer, the same lot of reagents and an identical lot of commercial control materials were used throughout the study. The intra-assay imprecision of the HIL indices was calculated (in percent values) by measuring each of the three Bio-Rad Liquichek Serum Indices (“Hemolysis”, “Icterus” and “Lipemia”) quality control materials in 20 consecutive runs, as recommended by the CLSI document EP05-A3 [18]. The performance goals of IQC for the HIL indices were instead estimated (in percent values) from inter-assay imprecision studies, by measuring the two fresh Bio-Rad Multi 1 and 3 IQC commercial materials and the three Bio-Rad Liquichek Serum Indices IQC materials at days 0, 1, 4, 8, 15, 22 and 29. The final performance goals were set as mean coefficient of variation (CV%) calculated using 3 standard deviations (SD) for each quality control material of each HIL index, as suggested by James Westgard (i.e., Westgard 1_3S_-rule) [19] and by the EFLM WG-PRE [14].

The significance of changes of HIL values throughout the study period was assessed with one-way analysis of variance (ANOVA) and direct comparison with performance goals (i.e., Westgard 1_3S_-rule) [19]. The statistical analysis was performed using Analyse-it (Analyse-it Software Ltd, Leeds, UK).

The material used in this study was obtained from anonymized routine lithium-heparin plasma samples once clinical chemistry testing had been completed. No additional tests were performed on routine samples other than those already ordered by the requesting physicians, so that patient’s informed consent was unnecessary. The study was approved by the local Institutional Review Board and Ethical Committee (University Hospital of Verona, Verona, Italy; SOPAV2, protocol number: 971CESC; date of approval: July 25, 2016).

## Results

The local intra-assay imprecision of HIL indices on Roche Cobas c702, estimated using Bio-Rad Liquichek Serum Indices IQC materials, was 1.1% for H-index, 2.0% for L-index and 1.7% for I-index, respectively (Table 2).

**Table 2:** Intra-assay imprecision (n = 20) of HIL (Hemolysis, H; Icterus, I; Lipaemia, L) indices on Roche Cobas c702, calculated using three liquid Bio-Rad Liquichek Serum Indices (“Hemolysis”, “Icterus” and “Lipemia”) quality control materials.

Parameter	Mean arbitrary value	Standard deviation	Coefficient of variation
H-index	101.3	1.1	1.1%
L-index	2124.5	43.0	2.0%
I-index	1029.5	17.1	1.7%

**Fig 1:**
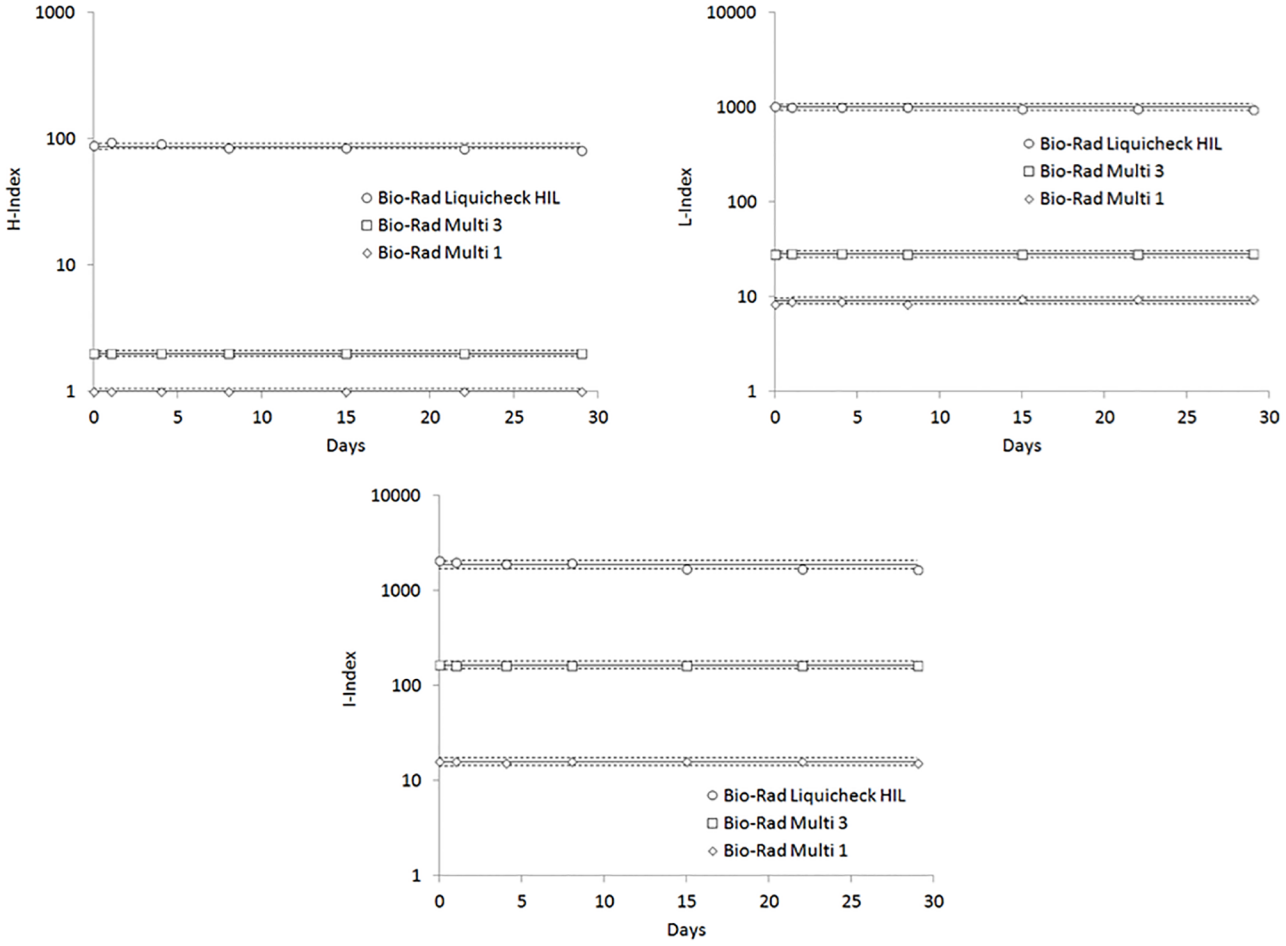
Instrument drift of HIL (Hemolysis, H; Icterus, I; Lipaemia, L) indices on Cobas c702 using Bio-Rad Multi levels 1 and 3 and Bio-Rad Liquichek Serum Indices (“Hemolysis”, “Icterus” and “Lipemia”). The continuous line is set at the target value, whilst the dotted lines define the performance goals.

The local inter-assay imprecision of HIL indices on Roche Cobas c702, calculated using commercial liquid IQC materials is shown in Table 3, and was comprised between 0–4.9% for H-index (mean imprecision, 1.6%; ANOVA, p = 0.468), 0.9–4.6% for L-index (mean imprecision, 2.5%; ANOVA, p = 0.495) and 0.6–7.9% for I-Index (mean imprecision, 3.3%; ANOVA, p = 0.460), respectively. These values were hence used to set local performance goals for accepting or rejecting data of in-house prepared IQC materials, by adapting the Westgard 1_3S_-rule (i.e., ≤4.9% for H-index, ≤7.6% for L-index and ≤9.9% for I-index, respectively) (Table 3 and Fig 1)19.

**Table 3:** Inter-assay imprecision (n = 7, over 29 days) and internal quality control (IQC) performance goals of HIL (Hemolysis, H; Icterus, I; Lipaemia, L) indices on Roche Cobas c702, calculated using liquid Bio-Rad Multi 1 and 3 quality control materials and liquid Bio-Rad Liquichek Serum Indices (“Hemolysis”, “Icterus” and “Lipemia”) quality control materials.

Parameter	Mean arbitrary value	Standard deviation	Coefficient of variation	Mean overall imprecision	IQC performance goals^a^
H-index				1.6%	≤4.9%
- Bio-Rad Multi 1	1	0	0%		
- Bio-Rad Multi 3	2	0	0%		
- Liquichek Serum Indices-H	87.1	4.3	4.9%		
L-index				2.5%	≤7.6%
- Bio-Rad Multi 1	9.1	0.4	4.6%		
- Bio-Rad Multi 3	28.2	0.2	0.9%		
- Liquichek Serum Indices-L	1000.9	21.7	2.2%		
I-index				3.3%	≤9.9%
- Bio-Rad Multi 1	15.9	0.2	1.4%		
- Bio-Rad Multi 3	165.1	1.0	0.6%		
- Liquichek Serum Indices-I	1880.3	147.8	7.9%		

^a^ mean coefficient of variation (CV%) calculated on 3 standard deviations (SD) of each quality control of each respective HIL index

**Fig 2:**
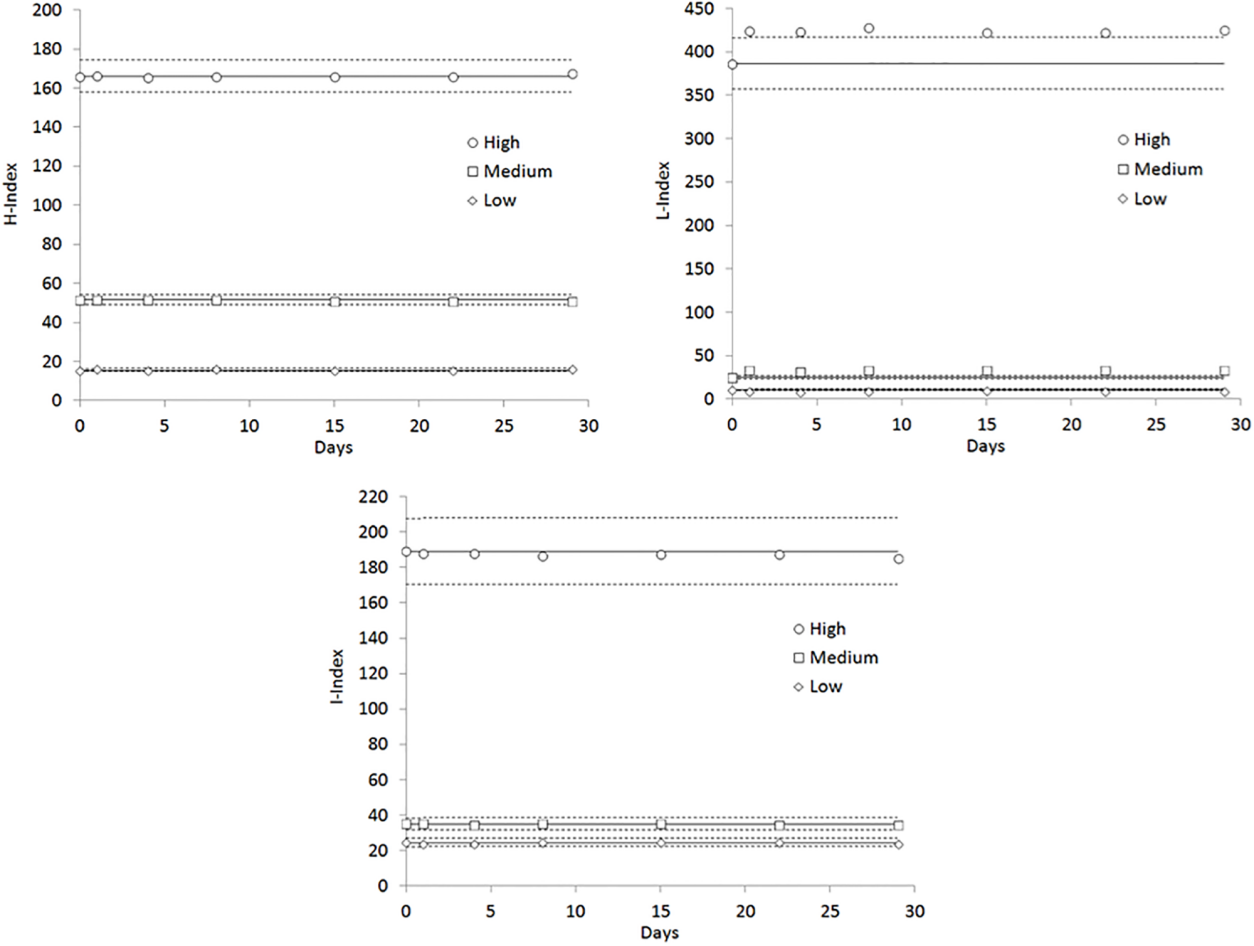
Stability of in-house prepared internal quality control (IQC) materials for quality assurance of HIL (Hemolysis, H; Icterus, I; Lipaemia, L) indices on Cobas c702, with target values set on fresh aliquots, on day 0. The continuous line is set at the target value, whilst the dotted lines define the performance goals.

The inter-assay imprecision of in-house prepared materials is shown in Fig 2 and Table 4.

**Table 4:** Inter-assay imprecision (n = 7 over 29 days) of HIL (Hemolysis, H; Icterus, I; Lipaemia, L) indices on Roche Cobas c702 using in-hose prepared frozen internal quality control (IQC) material, with target values set on the fresh plasma pools, on day 0.

Parameter	Mean arbitrary value	Standard deviation	Coefficient of variation	Mean overall imprecision
H-index				0.8%
- Level L	15.7	0.25	1.6%	
- Level M	51.3	0.25	0.5%	
- Level H	166.2	0.59	0.4%	
L-index				6.2%
- Level L	9.5	0.6	6.3%	
- Level M	32.1	2.9	9.1%	
- Level H	419.3	13.5	3.2%	
I-index				0.8%
- Level L	24.3	0.2	1.0%	
- Level M	34.8	0.2	0.7%	
- Level H	187.4	1.2	0.6%	

L, Low; M, Medium; H, High

**Fig 3:**
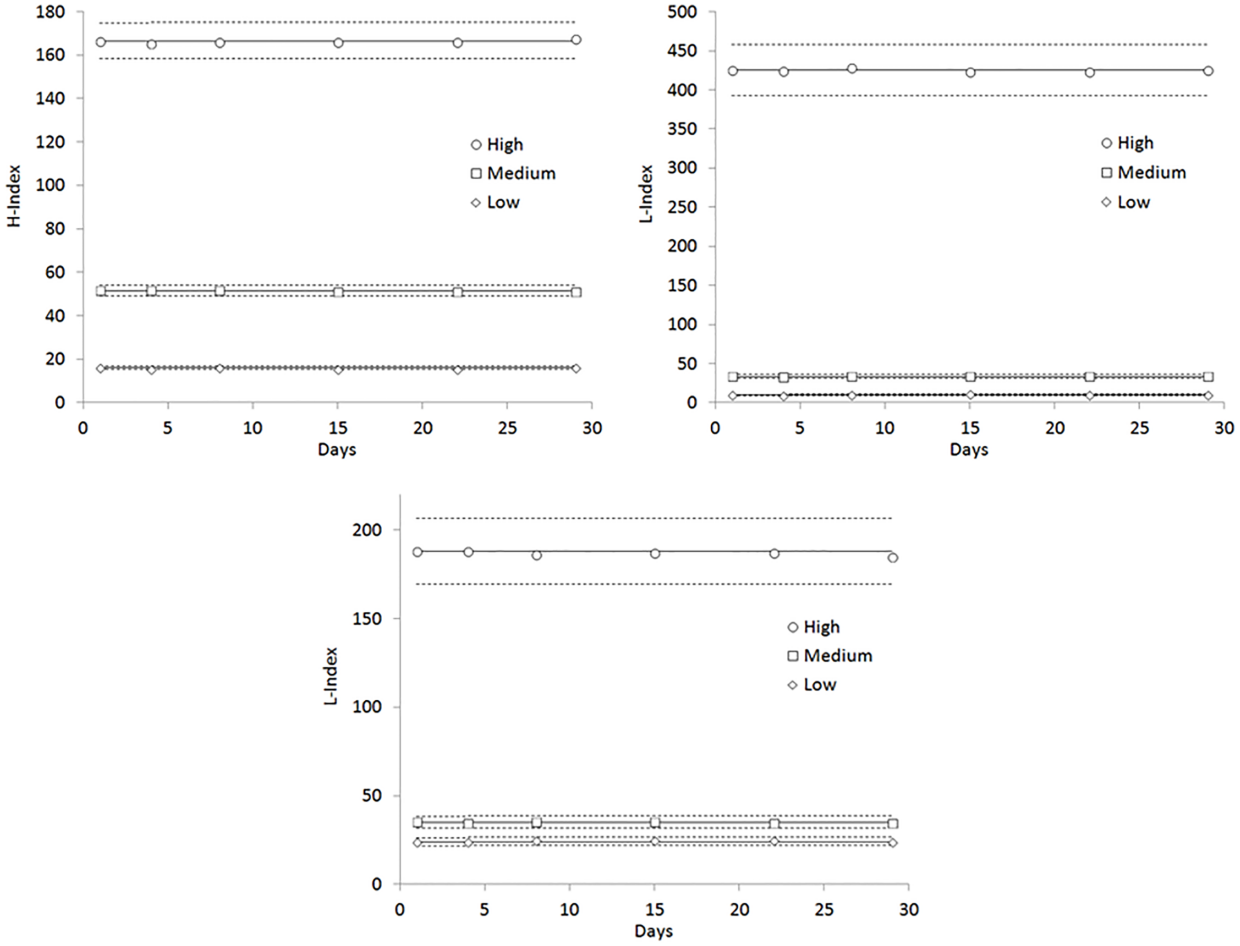
Stability of in-house prepared internal quality control (IQC) materials for quality assurance of HIL (Hemolysis, H; Icterus, I; Lipaemia, L) indices on Cobas c702, with target values set on the first frozen-thawed aliquot, on day 1. The continuous line is set at the target value, whilst the dotted lines define the performance goals.

When target values were set on fresh materials, all data generated with both H-index (ANOVA, p = 0.225) and I-index (ANOVA, p = 0.262) were comprised between their respective performance goals, whilst those of L-index (ANOVA, p = 0.225) were outside the acceptance limits, reflecting a significant variation of triglycerides and cholesterol values already occurring after the first freezing-thawing cycle (S1 Table). Unlike cholesterol and triglycerides, the values of both total and unconjugated bilirubin in frozen-thawed aliquots did not significantly differ from those obtained using fresh plasma (S1 Table). This evidence lead us to replace the target values obtained on fresh materials with those generated using the first frozen-thawed aliquot (day 1), as shown in Fig 3 and Table 5.

**Table 5:** Inter-assay imprecision (n = 6 over 28 days) of HIL (Hemolysis, H; Icterus, I; Lipaemia, L) indices on Roche Cobas c702 using in-hose prepared frozen internal quality control (IQC) material, with target values set on the first frozen-thawed aliquot, on day 1.

Parameter	Mean arbitrary value	Standard deviation	Coefficient of variation	Mean overall imprecision
H-index				0.8%
- Level L	15.8	0.3	1.6%	
- Level M	51.3	0.3	0.5%	
- Level H	166.3	0.6	0.4%	
L-index				2.2%
- Level L	9.3	0.5	5.1%	
- Level M	33.3	0.4	1.1%	
- Level H	424.8	1.7	0.4%	
I-index				0.8%
- Level L	24.3	0.3	1.0%	
- Level M	34.8	0.3	0.7%	
- Level H	187.1	1.1	0.6%	

L, Low; M, Medium; H, High

Interestingly, the adoption of target values measured in day 1 frozen-thawed aliquots allowed to maintain the inter-assay imprecision of L-index (ANOVA, p = 0.387) within its performance goals throughout the study period, with no substantial variation of inter-assay imprecision of both the H-index (ANOVA, p = 0.300) and I-index (p = 0.347) (Fig 3). In particular, the overall inter-assay imprecision was found to be virtually identical when target values were calculated on frozen-thawed aliquots compared to those calculated using fresh pools for both H-index (0.8% versus 0.8%) and I-index (0.8% versus 0.8%), whilst the overall inter-assay imprecision of L-index was found to be nearly 3-fold lower (2.2% versus 6.2%) (Tables 4 and 5).

Notably, when target values were defined on the first frozen-thawed aliquots, the overall inter-assay imprecision of in-house prepared materials was also found to be better than that calculated using commercial liquid IQC materials (H-index, 0.8% versus 1.6%; L-index, 2.2% versus 2.5%; I-index, 0.8% versus 3.3%).

## Discussion

Quality assurance is a mainstay in laboratory medicine, since it permits detecting mistakes and driving efforts to improve quality by implementing or revising standard operating procedures (SOP) [20]. This straightforward concept, involving the use of both IQC and EQA programs, has been successfully applied for decades to routine laboratory testing [21], and should then be broadened to all innovative tests that will become available for routine. The use of HIL indices for automatic assessment of sample quality has recently emerged as a virtually unavoidable practice for reliable identifying samples that may be unsuitable for laboratory testing due to the presence of high concentrations of some interfering substances such as cell-free hemoglobin, lipaemia and bilirubin [3,4]. The recent recommendations of the EFLM WG-PRE have endorsed the use of in-house prepared quality control materials, alone or in combination with commercial IQCs, for regular quality assurance of HIL indices [14]. The use of locally prepared IQCs was considered advantageous for many reasons, including cost minimization, better uniformity with biological material (i.e., serum or plasma), higher commutability and enhanced accuracy [11,12,13]. However, no evidence has been provided so far about the feasibility, validity and effectiveness of using in-house prepared quality control materials for systematic monitoring of HIL indices performance to the best of our knowledge.

The first important information emerged from our study is that the local intra-assay imprecision of HIL indices on Roche Cobas c702 CVs using commercial liquid IQC materials was globally comparable to that previously obtained by Nikolac Gabaj et al using a Roche Cobas c501 for H-index (1.1% versus 0.8–1.6%), was slightly higher for L-index (2.0% versus 0.4–1.2%), whist better results were obtained for I-index (1.7% versus 2.0–9.8%)^16^. The inter-assay imprecision calculated using commercial liquid IQC materials of H-index (0–4.9% versus 0.9–1.8%) and L-index (0.9–4.6% versus 0.5–1.9%) was slightly higher than that previously reported by Nikolac Gabaj et al^16^, whilst slightly better results were obtained for I-index (0.6–7.9% versus 2.0–11.3%). This inter-assay imprecision data was hence used to define the performance goals to be used for accepting or rejecting results generated using in-house prepared IQC materials (Table 3).

The second important finding of our study is that the use of in-house prepared IQC materials is a suitable practice for day-to-day monitoring of HIL indices performance. Interestingly, inter-assay imprecision of both H-index and I-index was satisfactory even when the target values of in-house prepared IQC materials were calculated using fresh plasma pools, whilst a significant bias was early observed for the L-index (i.e., starting from the first day of measurement of frozen-thawed aliquots) (Fig 2). Such a significant bias possibly reflects the unfavourable effect of the freezing-thawing cycle on lipids and lipoproteins, as previously identified in other studies [22,23], and then confirmed in our investigation (S1 Table). In particular, L-index data generated on frozen-thawed aliquots were all outside the acceptance limits set on fresh plasma (Fig 2), thus suggesting that the target values should be most conveniently defined on frozen-thawed rather than on fresh plasma pools. Strong support to this suggestion emerges from data shown in Fig 3 and Table 5, since the definition of target values on frozen-thawed plasma allowed obtaining higher ANOVA p-values (and thereby lower differences) for all HIL indices, the overall inter-assay imprecision was similar or even lower and the overall day-to-day imprecision of the L-index could then be brought back within its relative performance goals throughout the study period. In agreement with data earlier published by Petrova et al. [13], we also found that the overall inter-assay imprecision of all HIL indices was lower using in-house prepared materials than liquid commercial IQCs, thus supporting the notion that matrix-related bias can be reduced and commutability can be enhanced when commercial IQC materials are replaced by clinical patient samples [10,24]. Since regular analysis of commercial IQC materials would pose a substantial economic burden on the laboratory, the preparation of in-house IQC materials from routine samples will hence, al least partially, relief laboratory budgets from this extra-expenditure. Albeit we acknowledge that the number of measurements used in our study for assessing inter-assay imprecision and sample stability was limited, and was mostly based on single measurements, we reproduced routine laboratory practice for IQC assessment, also reflecting current CLSI recommendations about quality assessment of HIL indices [8]. Indeed, local laboratories may consider generating a larger number of data points for each tube and for more precisely assessing inter-assay imprecision and sample stability. Importantly, the quality in preparation of these materials in terms of reproducibility (batch-to-batch variation), comparability and trueness should be strictly guaranteed by the laboratory staff, by preparing plasma pools with fairly constant concentrations of interfering substances (i.e., hemoglobin, bilirubin and triglycerides), as those reported in Table 1. Moreover, each new batch of in-house IQC materials should be tested in parallel with the former batch, so that comparability and trueness of target values can be assured before the new IQC materials will be used for monitoring HIL performance in routine laboratory practice. Provided that a commutable batch of in-house IQC materials can be used, no change in matrix-related bias will be expected, as previously shown by Miller et al [10].

Although the HIL indices may not be subjected to the same strict requirements by regulatory and/or accrediting bodies as for other laboratory tests, routine verification of expected performance of these tests is strongly advocated by the CLSI [8]. A local approach, based on in-house IQC materials prepared from routine samples is one of the approaches suggested by the CLSI, thus implying that all clinical laboratories should take full legal responsibility of this action, due to the many important clinical decisions that may be undertaken on data generated, by the HIL indices [25]. Taken together, the results of this study would hence lead us to conclude that in-house IQC materials prepared from routine lithium-heparin plasma samples and stored frozen at -20°C for up to 1 month may be a more practical, cost-effective and even more technically suitable alternative (i.e., due to the use of a more uniform sample matrix) to commercial liquid IQC materials for purposes of HIL quality assurance.

## Supporting information

S1 Table Variation of triglycerides, total cholesterol, total and unconjugated bilirubin after the first freezing-thawing cycle. Significance of difference was assessed with one-way analysis of variance (ANOVA). L, Low; M, Medium; H, High.
(DOC)
