# Supplementary material for: Internal quality assurance of HIL indices on Roche Cobas c702
Source: PLoS One. 2018 Jul 6;13(7):e0200088. doi: 10.1371/journal.pone.0200088 (PMC6034854; doi:10.1371/journal.pone.0200088)
Supplement: S1 Table — L, Low; M, Medium; H, High. (DOC) [file pone.0200088.s001.doc]

**S1 Table. Variation of triglycerides, total cholesterol, total and unconjugated bilirubin after the first freezing-thawing cycle. Significance of difference was assessed with one-way analysis of variance (ANOVA).**

| **Parameters** |  | **Fresh plasma** |  | **Frozen-thawed plasma** |  | **p** |
| --- | --- | --- | --- | --- | --- | --- |
|  |  |  |  |  |  |  |
| Triglycerides (mmol/L) |  |  |  |  |  |  |
| - L-index L |  | 0.81 |  | 0.88 |  | 0.010 |
| - L-index M |  | 1.39 |  | 1.40 |  |
| - L-index H |  | 11.85 |  | 11.97 |  |
|  |  |  |  |  |  |  |
| Total cholesterol (mmol/L) |  |  |  |  |  |  |
| - L-index L |  | 4.68 |  | 4.61 |  | <0.001 |
| - L-index M |  | 4.49 |  | 4.34 |  |
| - L-index H |  | 4.36 |  | 4.24 |  |
|  |  |  |  |  |  |  |
| Total bilirubin (μmol/L) |  |  |  |  |  |  |
| - I-index L |  | 13.0 |  | 11.2 |  | 0.261 |
| - I-index M |  | 22.4 |  | 21.2 |  |
| - I-index H |  | 148.2 |  | 149.9 |  |
|  |  |  |  |  |  |  |
| Unconjugated bilirubin (μmol/L) |  |  |  |  |  |  |
| - I-index L |  | 4.7 |  | 4.7 |  | 0.069 |
| - I-index M |  | 13.8 |  | 13.7 |  |
| - I-index H |  | 117.7 |  | 115.2 |  |
|  |  |  |  |  |  |  |

L, Low; M, Medium; H, High
